# Exploratory analysis to predict pneumonitis during durvalumab consolidation therapy for patients with locally advanced non‐small cell lung cancer from proteomic profiling of circulating extracellular vesicles

**DOI:** 10.1111/1759-7714.15077

**Published:** 2023-08-24

**Authors:** Masahiro Torasawa, Hidehito Horinouchi, Shigehiro Yagishita, Hirofumi Utsumi, Keitaro Okuda, Daisuke Takekoshi, Saburo Ito, Hiroshi Wakui, Saori Murata, Sawako Kaku, Kae Okuma, Yuji Matsumoto, Yuki Shinno, Yusuke Okuma, Tatsuya Yoshida, Yasushi Goto, Noboru Yamamoto, Jun Araya, Yuichiro Ohe, Yu Fujita

**Affiliations:** ^1^ Department of Thoracic Oncology National Cancer Center Hospital Tokyo Japan; ^2^ Department of Respiratory Medicine Juntendo University Graduate School of Medicine Tokyo Japan; ^3^ Division of Molecular Pharmacology National Cancer Center Research Institute Tokyo Japan; ^4^ Division of Respiratory Diseases, Department of Internal Medicine The Jikei University School of Medicine Tokyo Japan; ^5^ Department of Diagnostic Radiology National Cancer Center Hospital Tokyo Japan; ^6^ Department of Radiation Oncology National Cancer Center Hospital Tokyo Japan; ^7^ Department of Experimental Therapeutics National Cancer Center Hospital Tokyo Japan; ^8^ Division of Next‐Generation Drug Development Research, Research Center for Medical Sciences The Jikei University School of Medicine Tokyo Japan

**Keywords:** chemoradiotherapy, durvalumab, extracellular vesicles, pneumonitis

## Abstract

**Background:**

Risk factors for predicting pneumonitis during durvalumab consolidation after chemoradiotherapy (CRT) in locally advanced non‐small cell lung cancer (LA‐NSCLC) are still lacking. Extracellular vesicles (EVs) play a crucial role in intercellular communication and are potential diagnostic tools for various diseases.

**Methods:**

We retrospectively collected predurvalumab treatment serum samples from patients treated with durvalumab for LA‐NSCLC, isolated EVs using anti‐CD9 and anti‐CD63 antibodies, and performed proteomic analyses. We examined EV proteins that could predict the development of symptomatic pneumonitis (SP) during durvalumab treatment. Potential EV‐protein biomarkers were validated in an independent cohort.

**Results:**

In the discovery cohort, 73 patients were included, 49 with asymptomatic pneumonitis (AP) and 24 with SP. Of the 5797 proteins detected in circulating EVs, 33 were significantly elevated (fold change [FC] > 1.5, *p* < 0.05) in the SP group, indicating enrichment of the nuclear factor kappa B (NF‐κB) pathway. Patients with high levels of EV‐RELA, an NF‐κB subunit, had a higher incidence of SP than those with low levels of EV‐RELA (53.8% vs. 13.4%, *p* = 0.0017). In the receiver operating characteristic analysis, EV‐RELA demonstrated a higher area under the curve (AUC) than lung V20 (0.76 vs. 0.62) and was identified as an independent risk factor in the multivariate logistic regression analysis (*p* = 0.008, odds ratio 7.72). Moreover, high EV‐RELA was also a predictor of SP in the validation cohort comprising 43 patients (AUC of 0.80).

**Conclusions:**

Circulating EV‐RELA may be a predictive marker for symptomatic pneumonitis in patients with LA‐NSCLC treated with durvalumab.

## Graphical abstract

We investigated the extracellular vesicle (EV) proteins that could potentially predict the onset of symptomatic pneumonitis (SP) during durvalumab treatment for patients with locally advanced NSCLC. The discovery cohort revealed significant nuclear factor‐κB (NF‐κB) pathway enrichment in SP patients. Notably, patients exhibiting high levels of EV‐RELA, a subunit of NF‐κB, experienced a higher incidence of SP. Further supporting this observation, this finding was validated in an independent cohort.

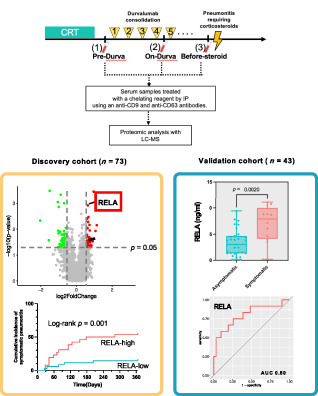

## INTRODUCTION

Approximately one‐third of patients with non‐small cell lung cancer (NSCLC) are diagnosed at a locally advanced stage of stage III.^1^ Platinum‐based chemotherapy with concurrent radiotherapy (CCRT) is the standard treatment for patients with unresectable stage III NSCLC. However, the expected 5‐year overall survival (OS) rate for patients with stage III unresectable NSCLC is 13%–36%,^2^ and the 10‐year OS rate for concurrent radiotherapy with carboplatin plus paclitaxel is 15.2% for those with unresectable stage III NSCLC^3^; therefore, a cure has been considered difficult for many patients. Recently, the PACIFIC trial (NCT02125461)^4^ demonstrated that up to 12 months of consolidation therapy with durvalumab, an anti‐programmed death ligand 1 (PD‐L1) antibody, significantly improved progression‐free survival (PFS) compared with placebo in patients with unresectable stage III NSCLC whose disease had not progressed following CCRT, establishing it as the new standard of care. The current data indicate an improved 5‐year survival rate of 42.9% (95% confidence interval [CI]: 38.2% to 47.4%).^5^

Pneumonitis, or interstitial lung disease, is a potentially fatal adverse event (AE) associated with both radiotherapy and immune checkpoint inhibitors (ICIs) for NSCLC. Symptomatic radiation pneumonitis occurs in 15%–40% of patients undergoing CCRT for NSCLC.^6^ Furthermore, ICIs exert their therapeutic effects by activating the immune system but can also cause immune‐related AEs (irAEs).^7^, ^8^ As the indications for CCRT and durvalumab increase, more patients might be at risk of developing pneumonitis. In fact, safety data from the PACIFIC trial revealed that pneumonitis was more common in the durvalumab group (33.9%) than in the placebo group (24.8%).^4^

To date, established risk factors for radiation pneumonitis^9^, ^10^, ^11^ or pneumonitis during durvalumab treatment after CCRT^12^, ^13^, ^14^, ^15^, ^16^ include interstitial pneumonitis, mean lung dose (MLD) (Gy), and lung parenchyma volume (V20) receiving 20 Gy, with MLD <20 Gy and V20 < 35% at the start of CCRT being recommended.^9^ However, apart from the clinical background, no biomarkers can currently predict severe pneumonitis during durvalumab treatment. Moreover, a subgroup analysis of the PACIFIC trial demonstrated that Asian patients were more susceptible to developing pneumonitis.^17^ Therefore, identifying biomarkers that can accurately predict which patients are at risk of developing pneumonitis and which require corticosteroid therapy is highly desirable, particularly in Asian populations.

Small extracellular vesicles (EVs), such as exosomes, are tiny particles <200 nm in diameter that are naturally secreted by all types of cells and present in various body fluids, including blood and urine.^18^, ^19^ Additionally, EVs contain a myriad of signaling molecules, including nucleic acids, proteins, and lipids, which may be involved in the physiological status of the parent cell information about the physiological state of the parent cell^20^ and potentially involved in intercellular communication.^21^ In the case of malignancy, EVs are involved in various processes underlying cancer progression, including inflammatory responses, angiogenesis, lymphopoiesis, cell proliferation, immunosuppression, metastasis, invasion, and epithelial‐mesenchymal transition.^22^ Furthermore, EV proteins have attracted attention as markers of the immune response, as recent studies have suggested that PD‐L1 in exosomes reflects the dynamic interaction between tumors and immune cells.^23^, ^24^ Therefore, we conducted a retrospective study to investigate the biomarkers that predict the development of symptomatic pneumonitis during durvalumab consolidation therapy using proteomic analysis of purified circulating EVs.

## METHODS

### Patient cohorts

For the discovery cohort, patients diagnosed with locally advanced NSCLC at a single institution (National Cancer Center Hospital, Tokyo, Japan) between January 2016 and February 2021 who had completed combination platinum‐based CCRT and were scheduled for durvalumab consolidation therapy, with available residual serum samples were included in this study. For the validation cohort, we enrolled patients diagnosed with locally advanced NSCLC at The Jikei University Hospital (Tokyo, Japan) between June 2020 and January 2023 who had completed combination platinum‐based CCRT and were scheduled for durvalumab consolidation therapy. Patients had no interstitial pneumonitis at CCRT initiation and a lung V20 of less than 35%. The study protocol was approved by the institutional review board (2019‐326) at National Cancer Center Hospital and (32‐056[10131]) at The Jikei University Hospital.

In this study, pneumonitis was defined as either radiation pneumonitis or durvalumab‐associated pneumonitis. Patients with pneumonitis of other etiologies, such as infection, were excluded. The diagnosis of pneumonitis in this study was based on the clinical judgment of the thoracic oncologist and was determined by the presence of radiographic and clinical evidence of pneumonitis following the initiation of durvalumab treatment. In the discovery cohort, two thoracic oncologists (MT and SM) and one independent radiologist (SK) determined the distribution of pneumonitis over the irradiated field. The pneumonitis grades were classified according to the Common Terminology Criteria for Adverse Events version 5.0.^26^ If no adverse event of pneumonitis was observed, it was defined as grade 0. The data cutoff date for the discovery and validation cohorts were October 31, 2022 and December 31, 2022, respectively.

We were able to collect the detailed clinical characteristics and treatment information from the patients' medical records in the discovery cohort. Regarding the dose parameters of lung toxicity, lung V5 and V20 were calculated. PD‐L1 expression was assessed by immunohistochemical staining (PD‐L1 immunohistochemistry 22C3, pharmDx, Dako/Agilent) of samples collected at diagnosis, and the PD‐L1 tumor percentage score was calculated as the percentage of viable tumor cells with PD‐L1 positive staining.^25^ PFS for durvalumab treatment was calculated from the first durvalumab dose until disease progression or death. In addition, patients who were alive without a recorded date of disease progression at the last follow‐up were censored. Completion of durvalumab treatment was defined as treatment completion at the discretion of the thoracic oncologist without tumor progression after approximately 1 year of durvalumab therapy (including a durvalumab withdrawal period due to pneumonitis).

### Blood sample collection

**FIGURE 1:**
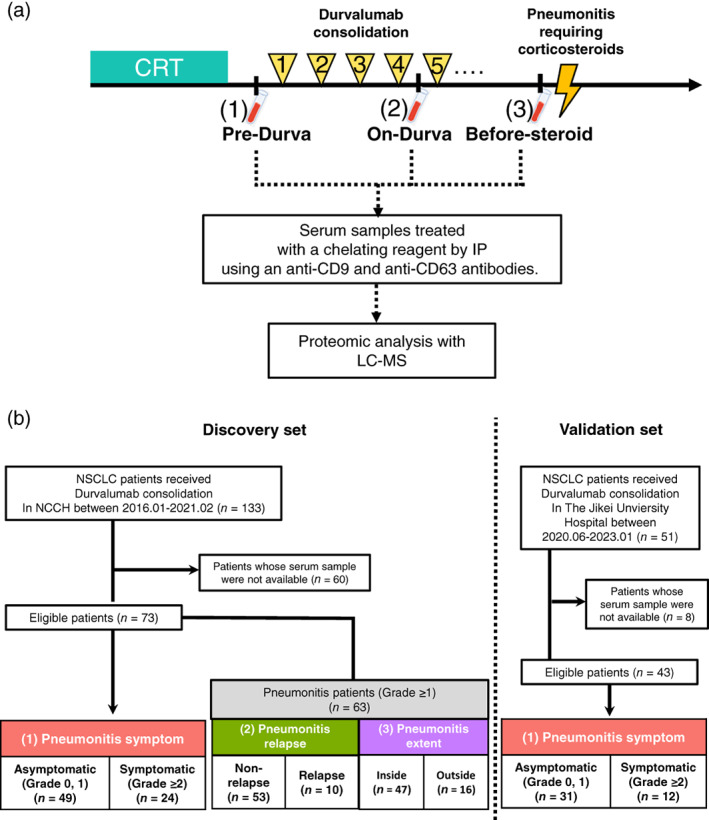
(a) Overview of the study. If the patient developed pneumonitis requiring corticosteroids before four doses of durvalumab, the timing of the “before‐steroid” blood collection is before the “on‐durva” samples. (b) Consort diagram and patient classification for this study. Durva, durvalumab; IP, immunoprecipitation; LC–MS, liquid chromatography–tandem mass spectrometry.

The primary outcome of this investigation was to explore biomarkers to predict symptomatic pneumonitis during durvalumab therapy using predurvalumab treatment of blood specimens. As a secondary outcome, we aimed to identify changes in EV protein expression as potential biomarkers during durvalumab treatment. For the discovery cohort, we utilized residual serum from patients treated with durvalumab at the three‐time points shown below; first (predurva): before the first dose of durvalumab and within 42 days after completion of CRT; second (on‐durva): after four doses of durvalumab; third (before‐steroid): if pneumonitis that requires corticosteroid administration develops, taken immediately before corticosteroid administration (Figure 1a). The serum samples were obtained from the National Cancer Center (Biobank, Japan). For the validation cohort, we utilized serum samples from patients treated only with durvalumab at the “predurva” time point. The serum samples in the validation cohort were obtained from The Jikei University Hospital.

### Comparison subsets

We categorized the overall cohort of patients into two groups based on the symptoms of pneumonitis, namely, asymptomatic (grade 0–1) and symptomatic (grade ≥2) groups, to identify potential EV protein biomarkers that could predict the occurrence of symptomatic pneumonitis. In addition, we explored EV proteins related to durvalumab‐induced pneumonitis, including the occurrence of multiple events of pneumonitis (e.g., exacerbations during corticosteroid tapering or after durvalumab rechallenge) or the progression of pneumonitis beyond the radiation field. Therefore, we further classified patients who developed pneumonitis (grade ≥1) into the following two groups based on two additional criteria: (1) relapse of pneumonitis, which was classified into non‐relapse (pneumonitis only occurred once) and relapse (pneumonitis occurred more than two events) groups and (2) extent of pneumonitis, which was categorized into inside (pneumonitis infiltrations inside the radiation field) and outside (pneumonitis infiltrations outside the radiation field) groups.

### Isolation of EVs and proteome analysis

For the discovery cohort, EVs were isolated from the collected serum samples using anti‐CD9 and anti‐CD63 antibodies (H.U. Group Research Institute), which are molecular markers of EVs.^18^ EVs isolated by immunoprecipitation were checked for correct purification using electron microscopy. The proteins contained in EVs were analyzed by proteome analysis using liquid chromatography–tandem mass spectrometry. The raw mass spectrometry data were uploaded in the public database (https://doi.org/10.6084/m9.figshare.23607051.v1). Furthermore, if potential EV‐proteins that predict symptomatic pneumonitis were identified in the discovery cohort, we investigated the predictive value of the EV‐protein candidate in the validation cohort. For the analysis, we isolated EVs from serum samples (predurva) by ultracentrifugation. Lysed EVs were analyzed for EV‐protein concentration by ELISA. Detailed methods are described in the Supplementary Materials.

### Discovery of candidate EV proteins and bioinformatics analysis

EV proteins with missing values in >20% of the samples (3157 EV proteins) were eliminated.^27^ EV proteins that were differentially expressed between the two groups based on the symptoms of pneumonitis (asymptomatic and symptomatic groups) were extracted. Among the EV proteins that were elevated in the symptomatic pneumonitis group, we narrowed down the candidate EV proteins associated with pneumonitis by considering protein–protein interactions among the proteins comprising the significantly enriched pathway. Pathway enrichment analysis was performed using the Curated Reactome option of the ShinyGO database (version 0.76.3),^28^ with the false discovery rate (FDR) set to 5%, and 20 pathways were identified. The protein–protein interaction network between the enriched EV proteins was visualized using STRING.^29^ The same analysis was performed on the remaining two subsets (relapse and extent of pneumonitis). Volcano plots and heatmaps were charted and analyzed using ImageGP.^30^

### Statistical analysis

Categorical and continuous variables are summarized descriptively using percentages and medians. The Mann–Whitney U and Fisher's exact tests were used to test for differences and associations between continuous and categorical variables, respectively. EV proteins with a 1.5 or greater fold change (FC) and *p* < 0.05, as determined by the Mann–Whitney U test, were considered significantly differentially expressed between the groups. Notably, as this study was an exploratory analysis, multiple hypotheses were not tested;^31^ however, FDR was considered in the pathway enrichment analysis, as described above. Moreover, associations between EV protein levels and symptomatic pneumonitis were evaluated using receiver operating characteristic (ROC) curves, and logistic regression analysis was performed to explore factors influencing the development of symptomatic pneumonitis during durvalumab treatment. For survival analysis, PFS was analyzed using the Kaplan–Meier method, and differences were compared using the log‐rank test (two‐sided). Hazard ratios (HRs) and corresponding 95% CIs were estimated using a Cox proportional regression model. Statistical significance was defined as *p* < 0.05. All statistical analyses were performed using R statistical programming language (version 4.2.1) and GraphPad Prism 9.0 (GraphPad).

## RESULTS

### Patient characteristics

Consort diagrams of this study are shown in Figure 1b. In the discovery cohort, 133 patients with locally advanced NSCLC received durvalumab consolidation therapy, of whom 73 had serum samples available and met the eligibility criteria. At the data cutoff date, 63 of these patients had developed pneumonitis, of which 49 (67.1%) and 24 (32.9%) were categorized as asymptomatic (grade 0, 1) and symptomatic (grade ≥2), respectively, (median follow‐up months was 33.8 months). Of the 63 patients who developed pneumonitis, 10 had more than two pneumonitis events, and 16 presented pneumonitis images that exceeded the initially planned irradiation field. The maximum pneumonitis grade attained and subsequent outcome of durvalumab treatment are shown in Figure S1. Among the 24 patients who contracted grade ≥2 pneumonitis, 13 were rechallenged with durvalumab after their recovery from pneumonitis, of which four experienced a relapse of pneumonitis. Additionally, 17 of 24 patients could not achieve the initially planned durvalumab treatment due to discontinuation (7 and 10 discontinued treatments due to progressive disease [PD] and pneumonitis, respectively).

The patient characteristics are presented in Table 1. All patients had a PS of 0–1, and nearly all of them involved in the study fulfilled the eligibility criteria for the PACIFIC trial.^4^ Notably, a larger proportion of patients in the symptomatic group had higher PD‐L1 expression (≥50%) than in the asymptomatic group (symptomatic vs. asymptomatic group: 62.5% vs. 26.5%, *p* = 0.005). Lung V5 and V20 tended to be higher in the symptomatic group than in the asymptomatic group; however, no statistical difference was observed.

**TABLE 1:** Patient characteristics in the discovery cohort.

Factor		Overall (*n* = 73)		Asymptomatic (*n* = 49)		Symptomatic (*n* = 24)		*p*‐value
Age, median [range]		64	[38, 83]	64.9	[38, 83]	63.5	[46, 78]	0.605
Sex *n*, (%)	Female	17	(23.3)	12	(24.5)	5	(20.8)	1
	Male	56	(76.7)	37	(75.5)	19	(79.2)	
Smoking history *n*, (%)	Smoker	63	(86.3)	42	(85.7)	21	(87.5)	1
	Never smoker	10	(13.7)	7	(14.3)	3	(12.5)	
Brinkmann Index, median [range]		800	[0, 2820]	690	[0, 2820]	900	[0, 2000]	0.212
Histology *n*, (%)	Adenocarcinoma	40	(54.8)	27	(55.1)	13	(54.2)	0.746
	Squamous	21	(28.8)	15	(30.6)	6	(25.0)	
	Other	12	(16.4)	7	(14.3)	5	(20.8)	
Clinical stage *n*, (%)	IIB	2	(2.7)	2	(4.1)	0	(0.0)	0.403
	IIIA	22	(30.1)	14	(28.6)	8	(33.3)	
	IIIB	34	(46.6)	20	(40.8)	14	(58.3)	
	IIIC	7	(9.6)	6	(12.2)	1	(4.2)	
	Recurrence (rTNM Stage III)	8	(11.0)	7	(14.3)	1	(4.2)	
Performance status *n*, (%)	0	35	(47.9)	25	(51.0)	10	(41.7)	0.469
	1	38	(52.1)	24	(49.0)	14	(58.3)	
Pulmonary emphysema (%)	Yes	31	(42.5)	23	(46.9)	8	(33.3)	0.32
	No	42	(57.5)	26	(53.1)	16	(66.7)	
Driver mutation,^a^ *n* (%)	Mutant	12	(16.4)	9	(18.4)	3	(12.5)	0.739
	Negative or unknown	61	(83.6)	40	(81.6)	21	87.5)	
PD‐L1, *n* (%)	<1%	23	(31.5)	19	(38.8)	4	(16.7)	0.017
	1%–49%	17	(23.3)	12	(24.5)	5	(20.8)	
	≥50%	28	(38.4)	13	(26.5)	15	(62.5)	
	Unknown	5	(6.8)	5	(10.2)	0	(0.0)	
CRT regimen, *n* (%)	CDDP + PEM	1	(1.4)	0	(0.0)	1	(4.2)	0.399
	CDDP + S‐1	1	(1.4)	1	(2.0)	0	(0.0)	
	CDDP + VNR	53	(72.6)	33	(67.3)	20	(83.3)	
	CDDP + ETP	1	(1.4)	1	(2.0)	0	(0.0)	
	Low dose CBDCA	17	(23.3)	14	(28.5)	3	(12.5)	
Radiation modality, *n* (%)	3D‐CRT	46	(63.0)	30	(61.2)	16	(66.7)	0.798
	VMAT	27	(37.0)	19	(38.8)	8	(33.3)	
Radiation dose *n*, (%)		60.0	[54.0, 66.0]	60.0	[54.0, 66.0]	60.0	[60.0, 66.0]	0.777
V5 median [range]		35.4	[14.8, 56.8]	34.2	[14.8, 56.8]	39.0	[15.4, 55.0]	0.162
V20 median [range]		21.1	[6.4, 34.5]	18.5	[6.4, 32.6]	23.22	[7.8, 34.5]	0.101

Abbreviations: 3D‐CRT, three dimensional conformal radiation therapy; CBDCA, carboplatin; CDDP, cisplatin; ETP, etoposide; PEM, pemetrexed; VMAT, volumetric modulated arc therapy; VNR, vinorelbine.

^a^ Driver mutation: any druggable mutations or fusions of *EGFR*, *ALK*, *ROS1*, *MET*, *RET* detected in clinical practice.

The patient demographic data of the validation cohort is presented in Table S1. This cohort comprised 43 patients who met the same criteria as the discovery cohort. There were no significant differences in clinical factors, including PD‐L1 expression, between the symptomatic and asymptomatic pneumonitis groups.

### Proteomic profiles of circulating EVs of asymptomatic and symptomatic groups in the discovery cohort

**FIGURE 2:**
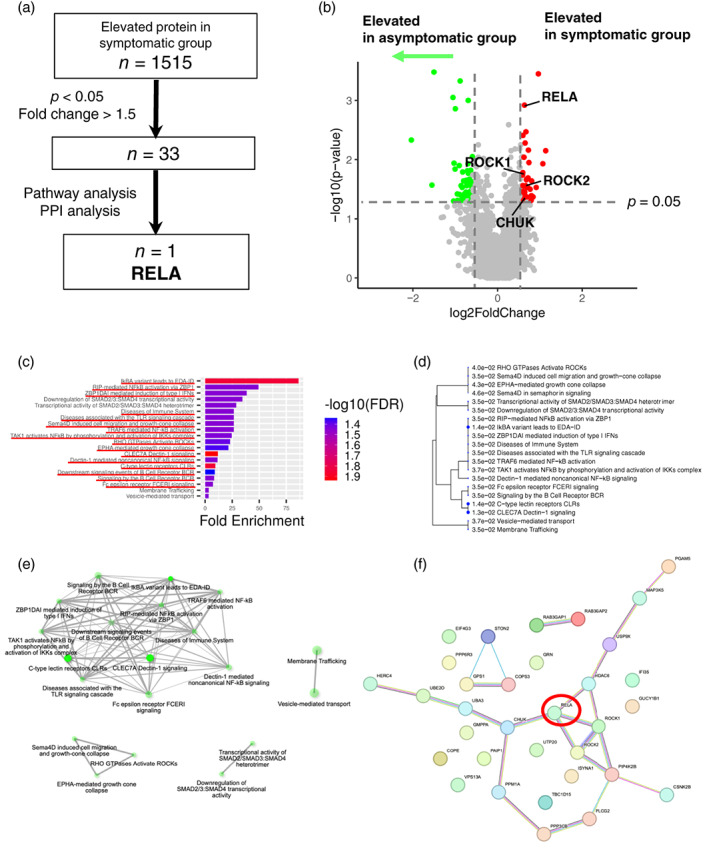
(a) Procedure for narrowing down candidate EV proteins in the discovery cohort. (b) Volcano plot representing EV‐proteins with elevated expression in patients with symptomatic and asymptomatic pneumonitis in serum samples before durvalumab administration in the discovery cohort. Annotated proteins involved in NF‐κB signaling. (c) Pathways enriched in EV‐proteins significantly elevated in patients with symptomatic pneumonitis in the discovery cohort. Pathways underlined in red are those related to NF‐κB. (d) A hierarchical clustering tree summarizes the correlation among enriched pathways in patients with symptomatic pneumonitis in the discovery cohort. (e) Network plot visualizing pathways enriched in patients with symptomatic pneumonitis in the discovery cohort. (f) Protein–protein interaction plot of EV proteins elevated in patients with symptomatic pneumonitis in the discovery cohort. RELA is highlighted with a red circle. EV, extracellular vesicle; NF‐κB, nuclear factor kappa B; PPI, protein–protein interaction.

We employed an immunoprecipitation (IP)‐based method that targets EV surface proteins to isolate specific EVs. IP in the presence of a chelating reagent improves the yield and purity of CD9 or CD63 positive EVs from serum samples. We confirmed that scanning electron microscope (SEM) images of the captured vesicles showed that vesicles measuring 50–100 nm are bound to the beads (Figure S2). Furthermore, the purity of the small EV marker CD9 or CD63 positive vesicles was higher than using the ultracentrifugation method (Figure S3). Therefore, IP is suitable for subsequent EV proteomics. We explored EV proteins that exhibited differential expression prior to durvalumab treatment initiation between symptomatic and asymptomatic groups to narrow down candidate biomarker proteins (Figure 2a). Of the 5796 proteins identified from circulating EVs, we incorporated 2639 in the analysis by eliminating those with missing values in over 20% of the samples (Table S2). Of the 1515 proteins elevated in the symptomatic group, 33 showed a 1.5‐fold or greater change compared with the asymptomatic group (*p* < 0.05) (Figure 2b). Details of the identified proteins and their corresponding coding genes are presented in Table 2.

**TABLE 2:** Information on the differentially expressed genes identified in the symptomatic pneumonitis group.

Gene	Protein identification	Protein description	*p*‐value	Fold change
MAP3K5	Q99683	Mitogen‐activated protein kinase kinase kinase 5	0.000354	1.950085
RELA	Q04206	Transcription factor p65	0.001198	1.554556
EIF4G3	O43432	Eukaryotic translation initiation factor 4 gamma 3	0.003403	1.592888
ISYNA1	Q9NPH2	Inositol‐3‐phosphate synthase 1	0.003924	1.516346
RAB3GAP2	Q9H2M9	Rab3 GTPase‐activating protein noncatalytic subunit	0.005259	1.571864
HDAC6	Q9UBN7	Histone deacetylase 6	0.00689	1.657665
GRN	P28799	Progranulin	0.007047	2.19951
PPP3CB	P16298	Serine/threonine‐protein phosphatase 2B catalytic subunit beta isoform	0.009137	1.541605
RAB3GAP1	Q15042	Rab3 GTPase‐activating protein catalytic subunit	0.011095	1.673053
PAIP1	Q9H074	Polyadenylate‐binding protein‐interacting protein 1	0.011855	2.100295
UTP20	O75691	Small subunit processome component 20 homolog	0.016596	1.512447
ROCK1	Q13464	Rho‐associated protein kinase 1	0.01826	1.507393
GPS1	Q13098	COP9 signalosome complex subunit 1	0.020222	1.646565
STON2	Q8WXE9	Stonin‐2	0.021582	1.609595
PIP4K2B	P78356	Phosphatidylinositol 5‐phosphate 4‐kinase type‐2 beta	0.022033	1.604991
HERC4	Q5GLZ8	Probable E3 ubiquitin‐protein ligase HERC4	0.022701	1.747497
ROCK2	O75116	Rho‐associated protein kinase 2	0.027269	1.58187
VPS13A	Q96RL7	Vacuolar protein sorting‐associated protein 13A	0.027317	1.514445
PGAM5	Q96HS1	Serine/threonine‐protein phosphatase PGAM5, mitochondrial	0.028344	1.54716
GMPPA	Q96IJ6	Mannose‐1‐phosphate guanyltransferase alpha	0.029417	1.619727
IFI35	P80217	Interferon‐induced 35 kDa protein	0.029538	1.883002
PPM1A	P35813	Protein phosphatase 1A	0.029824	1.637883
COPE	O14579	Coatomer subunit epsilon	0.031757	1.705304
GUCY1B1	Q02153	Guanylate cyclase soluble subunit beta‐1	0.034541	1.514396
TBC1D15	Q8TC07	TBC1 domain family member 15	0.035124	1.561395
UBA3	Q8TBC4	NEDD8‐activating enzyme E1 catalytic subunit	0.037486	1.551992
PPP6R3	Q5H9R7	Serine/threonine‐protein phosphatase 6 regulatory subunit 3	0.040415	1.741526
COPS3	Q9UNS2	COP9 signalosome complex subunit 3	0.042204	1.788008
USP9X	Q93008;O00507	Probable ubiquitin carboxyl‐terminal hydrolase FAF‐X	0.042204	1.615485
PLCG2	P16885	1‐phosphatidylinositol 4,5‐bisphosphate phosphodiesterase gamma‐2	0.042204	1.574997
CHUK	O15111	Inhibitor of nuclear factor kappa‐B kinase subunit alpha	0.044728	1.562851
CSNK2B	P67870	Casein kinase II subunit beta	0.049255	1.734686
UBE2O	Q9C0C9	(E3‐independent) E2 ubiquitin‐conjugating enzyme	0.049294	1.529425

Subsequently, we performed pathway enrichment analysis to investigate the shared characteristics of the aforementioned 33 EV proteins. We found that of the 20 significantly enriched (FDR <0.05) pathways in the symptomatic pneumonitis group, 16 were involved in nuclear factor kappa B (NF‐κB) signaling (Figure 2c–e). Pathways significantly enriched in the symptomatic groups are presented in Table S3, where RELA (p65) and CHUK (IKKα) were identified as NF‐κB signaling component proteins. RELA is a subunit that plays a central role in the NF‐κB family, and CHUK is an IκB kinase that leads to NF‐κB activation.^32^ In addition to NF‐κB signaling, the RhoA/Rho‐associated kinase (ROCK) pathway is associated with NF‐κB activation triggered by thrombin.^33^ Furthermore, protein–protein interaction analysis showed that RELA was interrelated with CHUK, ROCK1, and ROCK2 (Figure 2f), and we chose RELA as a candidate EV protein to predict symptomatic pneumonitis. To clarify the existence of RELA as a vesicular protein, we collected the EV or EV‐depleted serum fraction separated from the patient samples by ultracentrifugation. Notably, RELA was detectable in the CD9 positive EV fraction, but was undetectable in the EV‐depleted serum fraction positive for serum albumin (Figure S4). Taken together, EV‐RELA may be a novel biomarker for predicting symptomatic pneumonitis during durvalumab treatment.

### EV‐RELA and the occurrence of pneumonitis during durvalumab treatment in the discovery cohort

**FIGURE 3:**
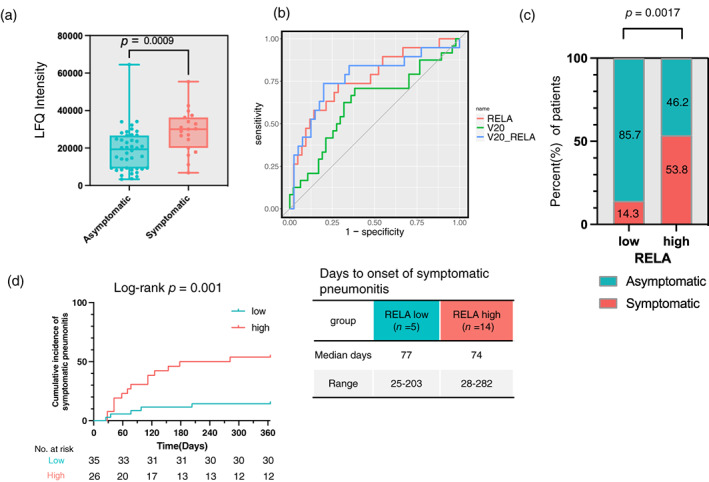
(a) Boxplot showing symptoms of pneumonitis and expression of EV‐RELA in the discovery cohort. (b) Receiver operating characteristic plot of risk factors for symptomatic pneumonitis development in the discovery cohort. (c) Percentage of asymptomatic and symptomatic pneumonitis in low and high RELA groups in the discovery cohort. (d) Kaplan–Meier plots showing the cumulative incidence of symptomatic pneumonitis in EV‐RELA subgroups in the discovery cohort.

We further investigated the association between the selected candidate proteins and pneumonitis. Our findings revealed that EV‐RELA levels were significantly higher in the symptomatic group than in the asymptomatic group (*p* = 0.001, Mann–Whitney U test) (Figure 3a). Additionally, EV‐RELA was assessed according to pneumonitis grade (grade 0/1/2/≥3). We observed no increase in protein expression with a worsening pneumonitis grade (Figure S5). However, the limited number of patients with grades 0 (*n* = 10) and ≥3 (*n* = 3) indicated insufficient statistical power for comparison.

Next, we conducted ROC analysis to determine the cutoff values for each candidate EV protein for predicting symptomatic pneumonitis (Figure 3b). For comparison, we also evaluated lung V20, a known risk factor for pneumonitis during durvalumab treatment, and found that its cutoff was 22 Gy with an area under the curve (AUC) of 0.62 (95% CI: 0.47–0.77). In contrast, RELA exhibited a higher AUC than V20 (RELA: cutoff 24 552, AUC, 0.76 [95% CI: 0.63–0.90]). Moreover, multivariate ROC analysis incorporating V20 and EV‐RELA as covariates demonstrated the highest predictive performance (AUC of 0.77 [95% CI: 0.63–0.91]) and significantly outperformed V20 in predicting symptomatic pneumonitis (*p* = 0.02). In the multivariate logistic regression analysis evaluating the risk factors for symptomatic pneumonitis, EV‐RELA was identified as a significant risk factor (RELA: *p* = 0.008, OR 7.72 [95% CI: 1.70–35.0]), whereas V20 and PD‐L1 expression exhibited a trend towards increased risk; however, they did not reach statistical significance (Table S4).

When patients were stratified into two cohorts using the cutoff values computed in this study, a significantly higher proportion of patients in the high RELA group demonstrated symptomatic pneumonitis (RELA: *p* = 0.0017 [Fisher's exact test], OR 6.75 [95% CI: 1.81–29.61]) (Figure 3c). Additionally, Figure 3d shows the cumulative incidence of developing grade ≥2 pneumonitis in the Kaplan–Meier curves. However, no difference was observed between EV‐RELA levels and the time to onset of symptomatic pneumonitis.

### RELA protein levels of the EVs derived from patients with durvalumab‐associated pneumonitis in the discovery cohort

**FIGURE 4:**
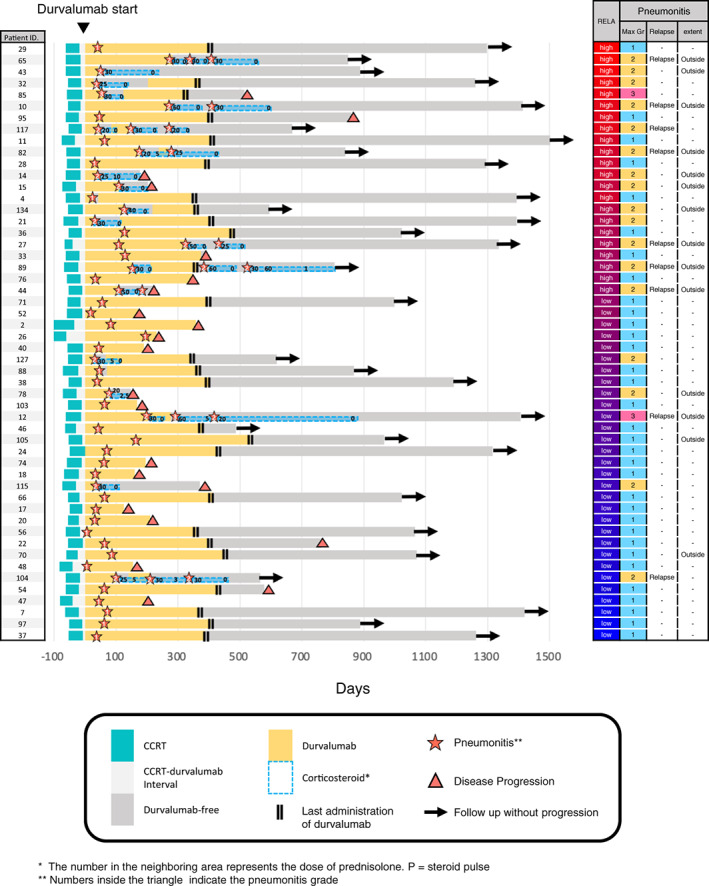
Swimmer plot showing the clinical course after durvalumab initiation in patients (*n* = 52) in whom EV‐RELA was measured in the discovery cohort. Patients are listed from top to bottom in order of EV‐RELA levels.

Durvalumab treatment may induce pneumonitis in patients who experience multiple episodes of pneumonitis or pneumonitis beyond the irradiation field. Therefore, we analyzed EV proteins that were differentially upregulated in these patients. Of the previously analyzed candidate proteins, EV‐RELA was significantly elevated in patients with pneumonitis extending beyond the irradiated field (*p* = 0.03, FC = 1.61) (Figure S6). Although the association between EV‐RELA and the development of more than two pneumonitis events was not statistically significant, RELA levels were higher in patients with multiple pneumonitis events (*p* = 0.08, FC = 1.35). Therefore, we illustrated the clinical course of patients (*n* = 52) who developed pneumonitis after durvalumab treatment initiation and measured their EV‐RELA levels (Figure 4). The patients were ranked based on their RELA levels, with the highest being at the top of the graph. Interestingly, in the high RELA group (*n* = 22, classified based on the ROC analysis cutoff), seven of 22 (31.8%) patients developed multiple events of pneumonitis, and 10 (45.5%) showed pneumonitis outside of the radiated field, with nine displaying bilateral lung infiltrations. In contrast, in the low RELA group, only two of 30 (6.7%) patients developed multiple events of pneumonitis, and four (13.3%) had pneumonitis that extended into the irradiated field, of which one showed bilateral lung shadows.

### Association of pneumonitis and durvalumab outcomes in the discovery cohort

Here, we investigated the association between pneumonitis and disease progression during durvalumab treatment. Initially, we evaluated the impact of factors, such as the symptoms of pneumonitis, and whether the pneumonitis image was detected outside the radiation field on PFS. Our findings revealed that neither the symptom of pneumonitis (asymptomatic vs. symptomatic: median PFS was not reached (NR), HR 0.49; 95% CI: 0.21–1.17; *p* = 0.49) nor the extension of the pneumonitis infiltrations (inside vs. outside: median PFS 29.5 vs. NR, HR 0.60; 95% CI: 0.26–1.39; *p* = 0.30) significantly impacted PFS (Figures S7A, B). Notably, significantly prolonged PFS was observed in patients with recurrent pneumonitis (nonrelapse vs. relapse: median PFS 23.5 vs. NR, HR 0.15; 95% CI: 0.06–0.39; *p* = 0.03) (Figure S7C). However, we found no significant association between PFS and EV‐RELA values (RELA low vs. RELA high: median PFS NR vs. 21.0, HR, 0.52; 95% CI: 0.25–1.09; *p* = 0.10) (Figure S7D).

### Changes in EV‐RELA protein levels following durvalumab initiation in the discovery cohort

Based on this analysis, EV‐RELA levels before durvalumab treatment are promising candidates for predicting durvalumab‐associated symptomatic (grade ≥2) pneumonitis. Therefore, to further investigate this, we evaluated the post‐durvalumab treatment trends in the EV‐RELA levels. Among patients with asymptomatic pneumonitis, we observed no significant difference in median RELA levels before and after four cycles of durvalumab treatment (Figure S8A), with some patients showing an increase in RELA levels and others showing a decrease (paired Wilcoxon test, *p* = 0.0827) (Figure S8B). In contrast, among patients with symptomatic pneumonitis, the median RELA level significantly decreased after four cycles of treatment (Figure S8C), and 13 of the 16 (81.3%) patients showed a decrease in RELA levels (paired Wilcoxon test, *p* = 0.0131) (Figure S8D). Notably, RELA levels also decreased with durvalumab treatment in patients receiving corticosteroids, as shown by the 3‐point trend (Figure S8E, F).

### Predictive value of EV‐RELA for predicting pneumonitis during durvalumab consolidation therapy in the validation cohort

**FIGURE 5:**
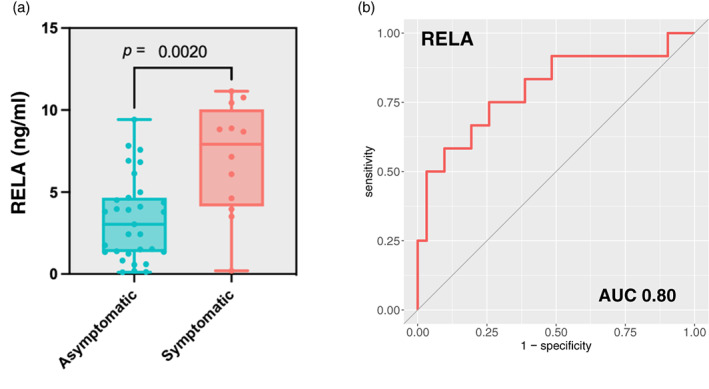
(a) Box plot showing symptoms of pneumonitis and expression of EV‐RELA in the validation cohort. (b) Receiver operating characteristic plot of EV‐RELA for symptomatic pneumonitis development in the validation cohort.

Finally, we investigated the predictive value of EV‐RELA in the validation cohort. We confirmed that EV‐RELA levels were significantly higher in the symptomatic group than in the asymptomatic group (*p* = 0.002, Mann–Whitney U test) (Figure 5a). In ROC analysis, the AUC of EV‐RELA was similar to the discovery cohort and showed high predictive power (cutoff 4.62 ng/mL, AUC of 0.80 [95% CI: 0.63–0.97]) (Figure 5b).

## DISCUSSION

This study assessed the circulating EV protein expression in patients without clinically detectable interstitial pneumonitis and with lung V20 < 35%. We found that RELA, which represents NF‐κB signaling, was elevated in patients who developed symptomatic pneumonitis after durvalumab treatment. Notably, EV‐RELA expression showed equal or greater predictive power for symptomatic pneumonitis than lung V20, a known risk factor for radiation‐induced or immunotherapy‐associated pneumonitis. Moreover, the expression of EV‐RELA also predicted symptomatic pneumonitis in the validation cohort.

EVs play a crucial role in intercellular communication and modulate the extracellular environment and immune response. As cancer cells secrete EVs containing cellular components that promote cancer, they are promising biomarkers for cancer diagnosis and therapeutic response evaluation.^34^ As EVs possess a lipid bilayer that enables stable transportation of their contents and potentially imitates the molecular profile of the originating cell, proteins obtained from EVs can serve as dependable, non‐invasive biomarkers that provide better insight into immune status than proteins acquired from plasma or serum. For example, several studies have demonstrated that exosome‐derived PD‐L1 is a better indicator of disease and immune activity than plasma‐derived soluble PD‐L1.^35^, ^36^ Although only a limited number of studies have been conducted on using EV proteins to predict drug therapy‐induced AEs,^37^ our research highlights the potential of EVs as biomarkers for predicting AEs.

NF‐κB signaling, which is elevated before durvalumab treatment in patients with symptomatic pneumonitis, is a master regulator of inflammation in lung endothelial cells.^33^ Irradiation directly damages DNA and generates reactive oxygen species, which releases various molecules (e.g., damage‐associated molecular pattern) and cytokines (e.g., interferon‐γ and transforming growth factor β) that promote inflammation and immune responses.^38^ These cytokines damage lung tissue by activating signaling pathways, such as the NF‐κB pathway. RELA is associated with acute lung injury and pulmonary fibrosis in human and mouse models.^39^, ^40^ ICI may also induce inflammation and immune responses in lung tissue, as they convert exhausted T cell phenotypes to active effector phenotypes, promoting cytokine production via T cell activation.^41^ The proinflammatory cytokine interleukin‐6, which is induced by the NF‐κB pathway, is reported to be an important mediator of irAEs in patients with NSCLC receiving ICIs.^42^ Overall, the elevation of EV‐RELA, which is a representative member of the NF‐κB family, in serum EVs before durvalumab initiation suggests that radiation‐induced activation of NF‐κB signaling triggers the onset of acute inflammation, even if no obvious pneumonitis is present during the durvalumab administration. In such patients, further T cell activation and cytokine upregulation by durvalumab would presumably induce pulmonary inflammation. In addition, EV‐RELA values in the asymptomatic pneumonitis group did not change significantly after the start of durvalumab treatment, whereas in the symptomatic pneumonitis group, there was a marked reduction in the EV‐RELA values after the therapy. Although no conclusion can be drawn regarding the clinical impact of protein expression changes, this observation may indicate a dynamic shift in the immune profile of the symptomatic group.

During durvalumab administration, both radiation and immunotherapy‐related pneumonitis may occur, and distinguishing between them can be challenging. A recent study proposed that they demonstrate distinct spatial features on computed tomography (CT) scans, with bilateral shadows and multiple lung lobes that are more typical of immunotherapy‐related than radiation.^43^ Remarkably, of the 24 patients who developed ≥2‐grade pneumonitis in this study, 16 had multiple pneumonitis events or extended pneumonitis outside the irradiated field. These multiple events of pneumonitis were either flare‐ups during corticosteroid tapering or after durvalumab was readministered once pneumonitis had subsided. In addition, 90% of pneumonitis cases that appeared outside the irradiated fields showed bilateral pneumonitis shadows. Notably, EV‐RELA was also associated with pneumonitis relapse and the development of pneumonitis outside the irradiated field, implying that EV‐RELA may be a marker for radiation pneumonitis and immunotherapy‐related AEs.

In the discovery cohort, patients with grade 2 or higher pneumonitis were found to have a greater likelihood of high tumor PD‐L1 expression. However, this trend was not confirmed in the validation cohort. A recent meta‐analysis reported that high PD‐L1 expression was associated with irAE appearance,^44^ and a retrospective study from a single center revealed that patients with high PD‐L1 expression suffered significantly more from ICI‐induced lung injury.^26^ Although NF‐κB upregulates tumor PD‐L1 expression, the level of RELA expression before radiotherapy initiation remains unknown. While the RELA high group had a higher frequency of patients with PD‐L1 ≥ 50% than the RELA low group, no significant difference was observed (33.3% vs. 50%, *p* = 0.28) (Figure S9A). Additionally, no correlation was found (Spearman correlation 0.20, *p* = 0.15) between the expression rates of tumor PD‐L1 and EV‐RELA (Figure S9B). Multivariate logistic regression analysis indicated that tumor PD‐L1 expression was not a significant risk factor for symptomatic pneumonitis, and further verification is required.

This study had some limitations. First, as this was a retrospective exploratory analysis conducted at a single center with a limited sample size, the selected proteins were not definitive biomarkers. However, our study identified EV proteins that may biologically explain the development of radiation and immunotherapy‐related pneumonitis, and similar results have been confirmed in the validation cohort. Therefore, these results should be prospectively validated in a larger population. Second, diagnosing pneumonitis is based on clinical judgment and may depend on the frequency of imaging evaluation. However, in this study, chest x‐ray examinations were performed every other time durvalumab was administered, and chest CT scans were performed every 3 to 6 months, even if the patients had no respiratory symptoms. Therefore, pneumonitis, including extension, was appropriately evaluated in this study. Third, as blood samples were not collected before the start of CCRT, whether the expression of candidate EV proteins increases due to irradiation remains unknown.

In conclusion, the evaluation of EV protein expression identified RELA as a potential predictive marker of symptomatic pneumonitis in patients treated with durvalumab after CCRT. Our study suggests that enhanced NF‐κB signaling after CCRT may be involved in the subsequent development of pneumonitis. Therefore, these findings should be prospectively validated in larger studies.

## AUTHOR CONTRIBUTIONS

**Masahiro Torasawa**: Investigation, data curation, methodology, software, formal analysis, resources, writing–original draft; writing–review and editing, visualization, final approval of the manuscript.

**Hidehito Horinouchi**: Investigation, data curation, methodology, writing–review and editing, supervision, final approval of the manuscript.

**Shigehiro Yagishita**: Investigation, data curation, methodology, writing–review and editing, final approval of the manuscript.

**Hirofumi Utsumi**: Investigation, resources, writing–review and editing, final approval of the manuscript.

**Keitaro Okuda**: Investigation, resources, writing–review and editing, final approval of the manuscript.

**Daisuke Takekoshi**: Investigation, resources, writing–review and editing, Final approval of the manuscript.

**Sabro Ito**: Investigation, resources, writing–review and editing, final approval of the manuscript.

**Hiroshi Wakui**: Investigation, resources, writing–review and editing, final approval of the manuscript.

**Saori Murata**: Investigation, writing–review and editing, final approval of the manuscript.

**Sawako Kaku**: Investigation, writing–review and editing, final approval of the manuscript.

**Kae Okuma**: Investigation, writing–review and editing, final approval of the manuscript.

**Yuji Matsumoto**: Investigation, writing–review and editing, final approval of the manuscript.

**Yuki Shinno**: Investigation, writing–review and editing, final approval of the manuscript.

**Yusuke Okuma**: Investigation, writing–review and editing, final approval of the manuscript.

**Tatsuya Yoshida**: Investigation, writing–review and editing, final approval of the manuscript.

**Yasushi Goto**: Investigation, writing–review and editing, final approval of the manuscript.

**Noboru Yamamoto**: Investigation, writing–review and editing, final approval of the manuscript.

**Jun Araya**: Investigation, resources, writing–review and editing, final approval of the manuscript.

**Yuichiro Ohe**: Investigation, writing–review and editing, final approval of the manuscript.

**Yu Fujita**: Conceptualization, methodology, resources, writing–review and editing, funding acquisition, project administration, final approval of the manuscript.

## CONFLICT OF INTEREST STATEMENT

Dr Torasawa has nothing to disclose. Dr. Horinouchi reports receiving personal fees from AstraZeneca.K.K during the conduct of the study; grants and personal fees from Chugai, Merck Sharp & Dohme, Novartis, Ono, and Roche; grants from AbbVie, Bristol‐Myers Squibb, Daiichi Sankyo, Genomic Health, Janssen, Merck Biopharma; and personal fees from Eli Lilly and Kyowa‐Kirin, outside of the submitted work. Dr Yagishita reports receiving grants from Nippon Boehringer Ingelheim, outside of the submitted work. Dr Utsumi has nothing to disclose. Dr Okuda has nothing to disclose. Dr Takekoshi has nothing to disclose. Dr Ito has nothing to disclose. Dr Wakui reports receiving personal fees from AstraZeneca.K.K during the conduct of the study.

Dr Murata has nothing to disclose. Dr Kaku has nothing to disclose. Dr Okuma has nothing to disclose. Dr Sinnno reports receiving personal fees from AstraZeneca.K.K during the conduct of the study; personal fees from Bristol‐Myers Squibb, Chugai, and Eli Lilly; grants and personal fees from Ono; and grants from Janssen and Japan Clinical Research Operations K.K. outside of the submitted work. Dr Matsumoto reports receiving personal fees from AstraZeneca.K.K during the conduct of the study; grants from Grant‐in‐Aid for Scientific Research on Innovative Areas, Hitachi, Ltd., and the National Cancer Center Research and Development Fund; grants and personal fees from Olympus; and personal fees from AMCO Inc., Chugai, COOK, Eli Lilly, Erbe Elektromedizin GmbH, Fujifilm, Novartis, and Thermo Fisher Scientific outside of the submitted work. Dr Okuma reports receiving personal fees from AstraZeneca.K.K during the conduct of the study; grants from AbbVie K.K. and Roche; and personal fees from Nippon Boehringer Ingelheim, Bristol‐Myers Squibb, Chugai Pharma Co. Ltd., Ely Lilly K.K., Ono Pharma Co. Ltd., Pfizer Taiho Pharma Co. Ltd., and Taiho Pharma Co. Ltd. outside of the submitted work. Dr Yoshida reports receiving personal fees from AstraZeneca.K.K during the conduct of the study; grants and personal fees from Amgen, Bristol‐Myers Squibb, Chugai, Merck Sharp & Dohme, Novartis, and Ono; grants from AbbVie, Daiichi Sankyo, and Takeda; and personal fees from ArcherDX, Eli Lilly, Roche, and Taiho outside of the submitted work. Dr Goto reports receiving personal fees from AstraZeneca.K.K during the conduct of the study; grants from AbbVie, AZK, Kyorin, and Preferred Network; grants and personal fees from Bristol‐Myers Squibb, Daiichi Sankyo, Eli Lilly, Novartis, Ono, and Pfizer; and personal fees from Boehringer Ingelheim, Chugai, Guardant Health Inc., Illumina, Merck Sharp & Dohme, Taiho, and Thermo Fisher outside of the submitted work. Dr. Yamamoto reports receiving personal fees from AstraZeneca.K.K during the conduct of the study; grants from Astellas, Bayer, Boehringer Ingelheim, Bristol‐Myers Squibb, Carna Biosciences, Chugai, Chiome Bioscience Inc., Daiichi Sankyo, Eisai, Eli Lilly, Genmab, GlaxoSmithKline, Janssen Pharma, Kyowa‐Hakko Kirin, Merck Sharp & Dohme, Novartis, Ono Pharmaceutical Co., Ltd., Otsuka, Pfizer, Quintiles, Shionogi, Sumitomo Dainippon, Takeda, and Taiho; and personal fees from Boehringer Ingelheim, Bristol‐Myers Squibb, Chugai, Cimic, Eisai, Lilly, Ono Pharmaceutical Co., Ltd., Otsuka, Pfizer, Sysmex, and Takeda, outside of the submitted work. Dr Araya receiving grants from Japanese Respiratory Foundation and Kowa company, LTD (campany x) outside of the submitted work. Dr Ohe reports receiving grants, personal fees, and nonfinancial support from AstraZeneca.K.K during the conduct of the study; personal fees from Amgen, AnHeeart Therapeutics Inc., Bayer, Boehringer Ingelheim, Bristol‐Myers Squibb, Celltrion, Chugai, Kyowa‐Hakko Kirin, Merck Sharp & Dohme, Nippon Kayaku, Ono Pharmaceutical Co., Ltd., Pfizer, and Taiho; grants and personal fees from Eli Lilly; grants and nonfinancial support from Kyorin; grants from Daiichi Sankyo, Dainippon‐Sumitomo, Janssen, Kissei, LOXO, Novartis, Takeda, and Taiho, outside of the submitted work. Dr Fujita reports receiving grants from AstraZeneca.K.K during the conduct of the study; grants from Preferred networks, Showa Denko Materials Co. Ltd., SHIBUYA Cooperation, H.U. Group Holdings, Inc., Merck Sharp & Dohme outside of the submitted work.

## Supporting information


**DATA S1.** Supporting Information.

**TABLE S2.** The list of proteins identified from the circulating EV proteomics.
