# Supplementary material for: Exploratory analysis to predict pneumonitis during durvalumab consolidation therapy for patients with locally advanced non‐small cell lung cancer from proteomic profiling of circulating extracellular vesicles
Source: Thorac Cancer. 2023 Aug 24;14(29):2909–23. doi: 10.1111/1759-7714.15077 (PMC10569905; doi:10.1111/1759-7714.15077)
Supplement: Supplementary file 1 — DATA S1. Supporting Information. [file TCA-14-2909-s001.docx]

**Supplementary Materials**

**Table of Contents**

1. **Isolation of EVs by immunoprecipitation**
2. **Scanning electron microscope images**
3. **Sample preparation for LC-MS**
4. **Proteomic analysis with LC-MS**
5. **Data-independent acquisition (DIA) analysis**
6. **Western blotting analysis of EVs isolated by ultracentrifugation**
7. **ELISA of EVs isolated by ultracentrifugation**
8. **Supplementary Table 1 and 3**
9. **Supplementary Figures (1–9)**

**1. Isolation of EVs by immunoprecipitation ~~Enrichment of EVs~~**

Serum EVs were captured using EViSTEP^TM^ EV isolation kit (H.U. Group Research Institute). In brief, 500 μL of serum was mixed with 500 μL of EViSTEP^TM^ reagent 1 (chelating-based reagent to enhance subsequent reaction), then the mixture was incubated with magnetic beads conjugated with anti-CD9 and anti-CD63 antibodies (H.U. Group Research Institute) at 37℃ for 60 min. Beads were then washed three times with EViSTEP^TM^ wash buffer. To minimize handling errors, the immunoprecipitation and wash steps were handled by an automated instrument, AutoEViS^TM^ (H.U. Group Research Institute). Lysis buffer (5 % SDS, 50 mM TEAB, pH 8.5) was added to washed beads and incubated for 5 min at room temperature. The samples were stored in -80℃.

**2. Scanning electron microscope images**

Regarding immunocaptured EVs, the antibodies conjugated to beads were added to an aliquot (500 µL) of the serum treated with PEVIA reagent (H.U. Group Research Institute), incubated on a rotator for 18 h at 4°C according to the manufacturing protocol, and washed three times with PBS. The samples were observed using a field emission SEM system (JSM-7500F, JEOL Ltd, Tokyo, Japan).

**3. Sample preparation for LC-MS**

The total protein concentration of lysed EVs was measured with Micro BCA Protein Assay kit (Thermo Fisher Scientific) to adjust the input protein amount to 18.5 ng or less. Sample preparation was performed following the instructions provided by the S-trap micro spin column (ProtiFi) with slight modifications. Samples were reduced with 20 mM dithiothreitol, and boiled at 95℃ for 10 minutes. After cooled down to room temperature, samples were alkylated with 40 mM iodoacetamide (Thermo Fisher Scientific), and they were incubated at room temperature for 30 minutes in the dark. Samples were then acidified with 2.5 µL of 12% phosphoric acid (FUJIFILM Wako Pure Chemical Corporation) to 25 µL samples, and then diluted with 165 µL of S-trap binding/wash buffer (100 mM TEAB, pH 7.1, Honeywell, in 90% methanol). The samples were left at room temperature for 5 minutes.

S-trap micro columns were equilibrated with 150 µL 0.2% formic acid (FA) in 50% acetonitrile, centrifuged at 4000g for 1 minute, and with 150 µL of S-trap binding/wash buffer followed by the centrifugation. After equilibration, samples were transferred to the S-trap columns and centrifuged at 4000g for 1 minute. The columns were washed with 200 µL of S-trap binding/wash buffer for four times with a 200 µL acetonitrile wash at the second time. The trapped proteins were subjected to the peptide digestion on the columns by 20 µL of Digestion buffer (50 mM TEAB containing 750 ng of Trypsin/Lys-C Mix, Promega) at 47℃ for 2 hours. The peptides were eluted with 40 µL of 50 mM TEAB, 40 µL of 0.2% FA, and 35 µL of 0.2% FA in 50% acetonitrile. Each fraction was pooled in the same collection tube. The eluants were freeze-dried and stored at -80℃ until further processing.

**4. Proteomic analysis with LC-MS**

Obtained peptides were reconstituted by 10 μl of water containing 0.1% formic acid (FA) (Fisher Chemical, Thermo Fisher Scientific). The amounts of peptides were determined by Pierce™ Quantitative Fluorometric Peptide Assay (Thermo Fisher Scientific). The samples were analyzed by EASY-nLC 1200 System (Thermo Fisher Scientific) coupled to Orbitrap Exploris 480 system (Thermo Fisher Scientific). Peptide sample (0.1 μg) was injected onto an Acclaim PepMap 100 trap column (75 μm × 2 cm, nanoViper C18 3 μm, 100Å, Thermo Fisher Scientific). The analytical column was C18 reverse-phase Aurora UHPLC Emitter Column with nanoZero (75 μm × 25 cm, Ion Opticks Pty Ltd) which was heated to 50℃ using ING ion source (AMR INCORPORATED). Nano pump flow rate was set to 300 nL/min with 70 min gradient, where the mobile phases were A (0.1% FA in water.) and B (95% acetonitrile and 5% water containing 0.1% FA.). The chromatography gradient was designed to provide a linear increase 0-0.5 min at 3% B, 0.5-32.5 min from 3% B to 16% B, 32.5-45 min from 16% B to 24% B, 45-54 min from 24% B to 33% B, 54-55 min from 33% B to 95% B, and wash for 15 min. The data-independent acquisition was performed in positive ion mode. The MS1 was performed the following parameters: m/z range: 500 to 860, resolution: 30,000, normalized AGC target: 300%, the maximum injection time: Auto, FAIMS CV voltage: -40. The MS2 was performed the following parameters: resolution: 30,000, normalized AGC target: 300%, the maximum injection time: 55 ms, collision energy: 30, isolation window: 6, no overlapping (divided into 60, cycle time, 3.3 seconds), FAIMS CV voltage: -40.

**5. Data-independent acquisition (DIA) analysis**

All DIA samples were analyzed using DIA-NN (version 1.8) which was set to search in silico spectral library. The spectral library was created by DIA-NN from SwissProt Human database FASTA (downloaded October 2021). The parameters for creating the spectral library are as follows: MS1 m/z range: 500-860, MS2 m/z range: 200-1800, fixed modification: carbamidomethyl (C), variable modification: acetyl (protein N-term), oxidation (M) and M excision (protein N-term). Two missed trypsin cleavages and two modifications per peptide were allowed. Library search by DIA-NN was performed the following parameters: Mass accuracy, MS1 accuracy, and Scan window: 0, Neural Network classifier: Double-pass mode, MBR: applied.

The top six fragments were used for peptide identification and quantification. MaxLFQ algorithm was used to calculate protein quantification^3^. The false discovery rate (FDR) was set to 1% at the peptide precursor and PG levels.

**6. Western blotting analysis of EVs isolated by ultracentrifugation**

EV protein expression of serum samples was analyzed by western blotting. To thoroughly remove cellular debris, the serum was filtered through a 0.22 μm pore-sized membrane filter (Stericup Quick Release Durapore, Merck Millipore). The 2.0 ml serum was diluted to 3.0 ml with particle-free PBS for ultracentrifugation. The tubes were centrifuged at 209,000 × g (44,200 rpm) for 45 min at 4 °C, using Beckman Coulter MLS-50 rotor. The pellets were washed with 5 mL of PBS by ultracentrifugation at 209,000 × g (44,200 rpm) using the MLS-50 rotor for 45 min at 4 °C and resuspended in PBS. EVs isolated from the serum were suspended in 100 μL M-PER regent (Thermo Fisher Scientific Inc). For each experiment, equal amounts of EV proteins were resolved by 4-20% gradient SDS-PAGE gels. Subsequently, the gels were transferred to a polyvinylidene difluoride (PVDF) membrane (Merck Millipore), and incubation with specific primary antibodies (mouse anti-RELA (p65) antibody (Cell Signaling Technology, #8242), mouse anti-Albumin antibody (Santa Cruz Biotechnology, #sc-271605), and mouse anti-CD9 antibody (Santa Cruz Biotechnology, #sc-59140)) was performed for 1 h at 37 °C. After washing several times with PBST, the membrane was incubated with anti-mouse IgG, HRP-linked secondary antibody (Cell Signaling Technology, #7076) followed by chemiluminescence detection (Thermo Fisher Scientific Inc, #34080) with the ChemiDocTM Touch Imaging System (BIO-RAD).

**7. ELISA of EVs isolated by ultracentrifugation**

For the validation set, candidate EV-protein expression of serum samples was analyzed by ELISA. After the EV isolation by ultracentrifugation as mentioned above, the EVs were suspended in 100 μL M-PER regent. The EV lysates were assayed for EV-protein by ELISA kit from RayBiotech according to the manufacturing protocol.

**References**

1 Cox J, Hein MY, Luber CA, Paron I, Nagaraj N, Mann M. Accurate proteome-wide label-free quantification by delayed normalization and maximal peptide ratio extraction, termed MaxLFQ. *Mol Cell Proteomics*. 2014;13(9):2513-2526.

**Supplementary Table 1. Patient characteristics in the validation set.**

| **Factor** | | **Overall** | | **Asymptomatic** | | **Symptomatic** | | ***p*-value** |
| --- | --- | --- | --- | --- | --- | --- | --- | --- |
|  |  | **(n =43)** | | **(n = 31)** | | **(n = 12)** | |  |
| **Age, median [range]** |  | 72 | [47, 85] | 72 | [47, 85] | 71.5 | [56, 80] | 0.924 |
| **Sex n, (%)** | Female | 12 | (27.9) | 9 | (29.0) | 3 | (25.0) | 1 |
|  | Male | 31 | (72.1) | 22 | (71.0) | 9 | (75.0) |  |
| **Smoking history n, (%)** | Smoker | 37 | (86.0) | 26 | (83.9) | 11 | (91.7) | 0.659 |
|  | Never smoker | 6 | (14.0) | 5 | (16.1) | 1 | (8.3) |  |
| **Brinkmann Index, median [range]** | | 800 | [0, 3000] | 700 | [0, 3000] | 1000 | [0, 2400] | 0.115 |
| **Histology n, (%)** | Adenocarcinoma | 19 | (44.2) | 14 | (45.2) | 5 | (41.7) | 0.812 |
|  | Squamous | 18 | (41.9) | 12 | (38.7) | 6 | (50.0) |  |
|  | Other | 6 | (14.0) | 5 | (16.1) | 1 | (8.3) |  |
| **Clinical stage n, (%)** | IIIA | 18 | (41.9) | 14 | (45.2) | 4 | (33.3) | 0.2 |
|  | IIIB | 18 | (41.9) | 10 | (32.3) | 8 | (66.7) |  |
|  | IIIC | 3 | (7.0) | 3 | (9.7) | 0 | (0.0) |  |
|  | Recurrence (rTNM Stage III) | 4 | (9.3) | 4 | (12.9) | 0 | (0.0) |  |
| **PD-L1, n (%)** | <1% | 7 | (16.3) | 5 | (16.1) | 2 | (16.7) | 0.444 |
|  | 1-49% | 5 | (11.6) | 2 | (6.5) | 3 | (25.0) |  |
|  | ≥50% | 13 | (30.2) | 10 | (32.3) | 3 | (25.0) |  |
|  | unknown | 18 | (41.9) | 14 | (45.2) | 4 | (33.3) |  |

**Supplementary Table 3. Information of identified enriched pathways in the symptomatic pneumonitis group in the discovery set (Reactome).**

| **Pathway** | **Enrichment**  **FDR** | **nGenes** | **Pathway**  **Genes** | **Fold**  **Enrichment** | **Genes** |
| --- | --- | --- | --- | --- | --- |
| **IkBA variant leads to EDA-ID** | 0.01405118 | 2 | 7 | 86.3387097 | RELA, CHUK |
| **RIP-mediated NFkB activation via ZBP1** | 0.03536431 | 2 | 17 | 49.3364055 | RELA, CHUK |
| **ZBP1DAI mediated induction of type I IFNs** | 0.03536431 | 2 | 24 | 38.3727599 | RELA, CHUK |
| **Downregulation of SMAD2/3:SMAD4 transcriptional activity** | 0.03536431 | 2 | 35 | 34.5354839 | PPM1A, USP9X |
| **Transcriptional activity of SMAD2/SMAD3:SMAD4 heterotrimer** | 0.03536431 | 2 | 58 | 28.7795699 | PPM1A, USP9X |
| **Diseases of Immune System** | 0.03536431 | 2 | 24 | 26.5657568 | RELA, CHUK |
| **Diseases associated with the TLR signaling cascade** | 0.03536431 | 2 | 24 | 26.5657568 | RELA, CHUK |
| **Sema4D induced cell migration and growth-cone collapse** | 0.03536431 | 2 | 25 | 26.5657568 | ROCK1, ROCK2 |
| **TRAF6 mediated NF-kB activation** | 0.03536431 | 2 | 32 | 26.5657568 | RELA, CHUK |
| **TAK1 activates NFkB by phosphorylation and activation of IKKs complex** | 0.03627336 | 2 | 32 | 24.6682028 | RELA, CHUK |
| **RHO GTPases Activate ROCKs** | 0.03938639 | 2 | 24 | 23.0236559 | ROCK1, ROCK2 |
| **EPHA-mediated growth cone collapse** | 0.04249047 | 2 | 49 | 21.5846774 | ROCK1, ROCK2 |
| **CLEC7A Dectin-1 signaling** | 0.01154283 | 5 | 107 | 11.8272205 | PPP3CB, UBA3, RELA, PLCG2, CHUK |
| **Dectin-1 mediated noncanonical NF-kB signaling** | 0.03536431 | 3 | 65 | 11.511828 | UBA3, RELA, CHUK |
| **C-type lectin receptors CLRs** | 0.01405118 | 5 | 151 | 9.48777029 | PPP3CB, UBA3, RELA, PLCG2, CHUK |
| **Downstream signaling events of B Cell Receptor BCR** | 0.04542254 | 3 | 97 | 8.93159066 | PPP3CB, RELA, CHUK |
| **Signaling by the B Cell Receptor BCR** | 0.03536431 | 4 | 260 | 8.42328875 | PPP3CB, RELA, PLCG2, CHUK |
| **Fc epsilon receptor FCERI signaling** | 0.03536431 | 4 | 272 | 7.34797529 | PPP3CB, RELA, PLCG2, CHUK |
| **Membrane Trafficking** | 0.03536431 | 8 | 671 | 3.39415075 | COPE, PPP6R3, RAB3GAP1, RAB3GAP2, TBC1D15, STON2, COPS3,  GPS1 |
| **Vesicle-mediated transport** | 0.03627336 | 8 | 782 | 3.15392547 | COPE, PPP6R3, RAB3GAP1, RAB3GAP2, TBC1D15, STON2, COPS3, GPS1 |

**Supplementary Table 4. Logistic regression analysis for the risk factor of symptomatic pneumonitis in the discovery set.**

| **Characteristics** | **Risk factors** | **Univariate** | | | **Multivariate** | | |
| --- | --- | --- | --- | --- | --- | --- | --- |
|  |  | **OR** | **95%CI** | **p-value** | **OR** | **95%CI** | **p-value** |
| Age | ≥65 yr (vs. <65 yr) | 0.81 | 0.31-2.16 | 0.68 |  |  |  |
| Sex | Male (vs. Female) | 1.23 | 0.38-4.01 | 0.73 |  |  |  |
| Smoking history | Current/Ex-Smoker (vs. Never smoker) | 1.17 | 0.27-4.98 | 0.84 |  |  |  |
| V20 | ≥22% (vs. <22%) | 3.53 | 1.25-9.95 | 0.02* | 2.01 | 0.54-7.40 | 0.30 |
| Radiation modality | VMAT (vs. 3D-CRT) | 0.79 | 0.28-2.20 | 0.65 |  |  |  |
| PD-L1 expression | ≥50% (vs. <50%) | 3.97 | 1.39-11.40 | 0.01* | 2.84 | 0.78-10.30 | 0.11 |
| RELA | High (vs. Low) | 7.00 | 2.06-23.70 | 0.002** | 5.97 | 1.61-22.10 | 0.008** |

*p< 0.05, ** p< 0.01
